# Radical Hemithoracic Radiotherapy Induces Systemic Metabolomics Changes That Are Associated with the Clinical Outcome of Malignant Pleural Mesothelioma Patients

**DOI:** 10.3390/cancers13030508

**Published:** 2021-01-29

**Authors:** Emanuela Di Gregorio, Gianmaria Miolo, Asia Saorin, Elena Muraro, Michela Cangemi, Alberto Revelant, Emilio Minatel, Marco Trovò, Agostino Steffan, Giuseppe Corona

**Affiliations:** 1 Immunopathology and Cancer Biomarkers Unit, Centro di Riferimento Oncologico di Aviano (CRO), IRCCS, 33081 Aviano, Italy; emanuela.digregorio@cro.it (E.D.G.); asia.saorin@cro.it (A.S.); emuraro@cro.it (E.M.); michela.cangemi@cro.it (M.C.); asteffan@cro.it (A.S.); 2 Medical Oncology and Cancer Prevention Unit, Centro di Riferimento Oncologico di Aviano (CRO), IRCCS, 33081 Aviano, Italy; gmiolo@cro.it; 3 Radiation Oncology Department, Centro di Riferimento Oncologico di Aviano (CRO), IRCCS, 33081 Aviano, Italy; alberto.revelant@cro.it (A.R.); eminatel@cro.it (E.M.); 4 Radiation Oncology Department, Azienda Sanitaria Integrata, 33100 Udine, Italy; marco.trovo@asufc.sanita.fvg.it

**Keywords:** metabolomics, mesothelioma, radiotherapy, biomarkers, cancers

## Abstract

**Simple Summary:**

Radical hemithoracic radiotherapy represents a promising new advance in the field of radiation oncology and encouraging results have been achieved in the treatment of malignant pleural mesothelioma patients. This study showed that this radiotherapy modality produces significant changes in serum metabolomics profile mainly affecting arginine and polyamine biosynthesis pathways. Interestingly, individual metabolomics alterations were found associated with the clinical overall survival outcome of the radiotherapy treatment. These results highlight metabolomics profile analysis as a powerful prognostic tool useful to better understand the mechanisms underlying the interpatients variability and to identify patients who may receive the best benefit from this specific radiotherapy treatment.

**Abstract:**

Radical hemithoracic radiotherapy (RHRT) represents an advanced therapeutic option able to improve overall survival of malignant pleural mesothelioma patients. This study aims to investigate the systemic effects of this radiotherapy modality on the serum metabolome and their potential implications in determining the individual clinical outcome. Nineteen patients undergoing RHRT at the dose of 50 Gy in 25 fractions were enrolled. Serum targeted metabolomics profiles were investigated at baseline and the end of radiotherapy by liquid chromatography and tandem mass spectrometry. Univariate and multivariate OPLS-DA analyses were applied to study the serum metabolomics changes induced by RHRT while PLS regression analysis to evaluate the association between such changes and overall survival. RHRT was found to affect almost all investigated metabolites classes, in particular, the amino acids citrulline and taurine, the C14, C18:1 and C18:2 acyl-carnitines as well as the unsaturated long chain phosphatidylcholines PC ae 42:5, PC ae 44:5 and PC ae 44:6 were significantly decreased. The enrichment analysis showed arginine metabolism and the polyamine biosynthesis as the most perturbed pathways. Moreover, specific metabolic changes encompassing the amino acids and acyl-carnitines resulted in association with the clinical outcome accounting for about 60% of the interpatients overall survival variability. This study highlighted that RHRT can induce profound systemic metabolic effects some of which may have a significant prognostic value. The integration of metabolomics in the clinical assessment of the malignant pleural mesothelioma could be useful to better identify the patients who can achieve the best benefit from the RHRT treatment.

## 1. Introduction

Malignant pleural mesothelioma (MPM) is a rare primary carcinoma originating from the pleural cavity, strongly linked to asbestos exposure [1]. The long latency after exposure and its characteristics of invasiveness and high aggressiveness contribute to make MPM a silent and invariable fatal disease with a median survival of less than 1 year when untreated [2]. The trimodal therapeutic approach that combines surgery, chemotherapy, and sequential radiotherapy (RT) represents the mainstream of current therapeutic protocols for MPM [3,4]. Over the last decades, RT technology has evolved [5], and the intensity-modulated radiation therapy (IMRT) has become one of the most interesting advance allowing the delivery of highly conformal radiation doses to the whole hemithorax limiting the normal tissue exposure. In MPM patients, this new RT modality referred as radical hemithoracic radiotherapy (RHRT) is delivered with a curative intent. However, despite its potential, its wide application is still debated especially for its possible severe toxicity [6], even if recent clinical investigations have shown encouraging results in enhancing patients’ survival with acceptable toxicity [7,8,9]. Despite the relevant overall survival gain, the clinical outcome of the RHRT was very heterogeneous among patients and there is an urgent need for prognostic biomarkers to guide clinical decision-making and to tailor the RHRT treatment. The knowledge of the molecular mechanisms involved in tumour and normal tissue response to RT has retained an important footstep to improve the efficacy of the treatment through the identification of specific molecular signatures useful to recognize patients who may achieve the best benefit from RT. In order to get more insight into the role of RHRT in the treatment of MPM patients, we investigated the host response to this specific treatment evaluating the systemic metabolic changes by the application of metabolomics and searched for potential new prognostic biomarkers.

Metabolomics is a rapidly advancing field that aims to characterize the concentration changes of all metabolites (<1 KDa) present in biological fluids or tissues [10]. The metabolomics profile describes the biochemical events occurring in an organism and reflects the complex interactions among age, sex, gene transcription, protein expression, physio-pathological conditions, and environmental effects as well as chemical or physical interventions such as the RT [11,12]. The radiation treatments may induce whole-body responses that can be mirrored and observed at the blood metabolome level. Hence, the blood metabolites composition represents a hypothetical source of biomarkers and the understanding of how metabolites and their concentrations change under RT interventions may allow the discovery of potential biomarkers for RT efficacy and toxicity. The effect of anticancer drug treatments on local and systemic metabolism have been widely investigated in different cancer types by the metabolomics tool [13,14,15,16]. Nevertheless, only a few broad-based metabolomics studies have been so far reported about the effects of RT on the host system [17,18,19,20,21,22,23] and none in the specific MPM field.

In attempt to fill this gap, this study aims to investigate the RHRT effects on the systemic metabolism by the analysis of changes in serum metabolomics profiles consequent to the treatment. The investigation provides new insights on the host biochemical alterations induced by the RHRT treatment and on their potential role in determining the individual clinical outcome. The results of this explorative translational investigation indicate that RHRT can produce profound effects on the serum metabolomics profile engaging amino acids and lipids metabolic pathways that could be relevant to establish the effective clinical benefit of the treatment.

## 2. Results

### 2.1. Demographic and Clinical Baseline Patients’ Characteristics

This translational study investigated 19 nonmetastatic MPM patients who underwent RHRT treatment consisting of 50 Gy in 25 fractions with a simultaneous integrated boost of 60 Gy in residual active disease. The clinical and demographic characteristics of the 19 MPM patients are reported in Table 1. The median age of the patients was 70 years (range: 33–79) with a great prevalence of male patients (89%). At baseline, 31% of patients presented adequate clinical conditions reporting an ECOG performance status (PS) score of 0, the majority of patients (53%) presented a PS score of 1 and only 16% had a PS score of 2. At the diagnosis, 95% of the MPM tumours had an epithelioid origin, while only 5% showed a biphasic histotype. Stage I–II characterized 47% of tumours, while the remaining 53% were classified as stage III–IV. The majority of the patients underwent previously nonradical surgical intervention for diagnostic purposes as biopsy (63%), and lung-sparing surgery pleurectomy/decortication (26%) or decortication (11%) leaving gross residual disease. All patients received systemic pharmacological treatment based on the pemetrexed and cisplatin chemotherapy. The RHRT was administered 4–6 weeks from the chemotherapy treatment.

The baseline characteristics of a reference group consisted of 15 MPM patients treated with standard palliative local RT (LRT). They are reported in Table S2. This reference group was characterized by superimposable demographic and clinical characteristics and underwent sparing surgery and chemotherapy treatment analogously to the RHRT group.

### 2.2. RHRT Effect on Serum Metabolome

**Figure 1:**
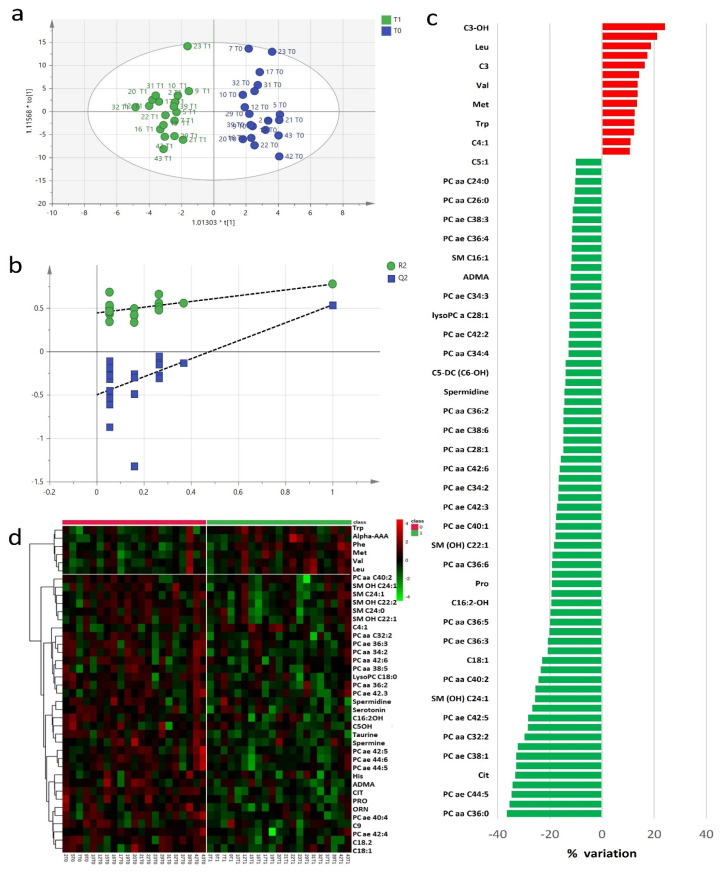
Orthogonal partial least squares discriminant analysis (OPLS-DA) score plot discriminated serum metabolomics profiles (*n* = 19) at baseline (T_0_, blue) and post-radical hemithoracic radiotherapy (RHRT) (T_1_, green) (**a**). Internal validation by permutation test showed R^2^ (green) and Q^2^ (blue) values from the permuted models (bottom left) significantly lower than the corresponding original model (top right) (**b**). Percentage variations of metabolites altered by RHRT (**c**). Heat map plot of the significantly changed serum metabolites between T_0_ samples (left) and T_1_ samples (right) ranked by *t*-test. Metabolites significantly decreased were in green, while metabolites significantly increased were in red. The brightness of the colour corresponded to the magnitude of the difference with the mean value (**d**).

The study of baseline and post-RHRT serum metabolomics profiles aimed to investigate the complex biochemical effects that RT may induce in the host. A targeted serum profile of 188 metabolites covering wide biochemical metabolic pathways was considered for this investigation (Table S1). Twenty-seven metabolites showed concentrations lower than the limit of detection and were excluded from further statistical analyses. Exploratory data analysis performed using principal component analysis (PCA) (Figure S1) did not detect any outliers. The metabolomics profile at baseline and after RHRT resulted homogeneous without any clusters of patients associated with the different diagnostic intervention as well as patients’ outliers. However, when the metabolomics profile at baseline and post-RHRT where compared, the PCA model explained only 22% of total variance and did not allow to characterize differences, supporting the application of supervised orthogonal partial least squares discriminant analysis (OPLS-DA) approach. Multivariate OPLS-DA model clearly differentiated the baseline serum metabolomic profiles from those post-RHRT with a significant discrimination power (Figure 1a) (*p* = 0.007, CV-ANOVA). OPLS-DA model showed good performance when internal leave-one-out cross-validation (LOOCV) was assessed (R^2^ = 0.77, Q^2^ = 0.54) without any potential risk of over-fitting verified by permutation test (Figure 1b). The extent of the RHRT effects on the serum metabolomics profile was estimated by determining the relative percentage of variation (Δ%) of serum concentrations of the metabolites with VIP > 1 that most contribute to the OPLS-DA model (Figure 1c). After RHRT, the 52% of investigated metabolites showed a serum variation of ≥10%; among them, 14 were upregulated, while 69 were downregulated. All these changes were not found associated with the gross residual disease indicating that they could not be attributed to the tumour extent but likely to host metabolic response.

The wide decrease in serum metabolites concentrations after RHRT is clearly indicated by the heat map for the selected statistically significant metabolites (Figure 1d). Only a small set of metabolites, mainly belonging to the amino acids class, significantly increased after irradiation. It included the aromatic AA phenylalanine and tryptophan, the branched amino acids (BCAA) valine and leucine and alpha aminoadipic acid, methionine and carnitine. Conversely, almost all the phospholipids, encompassing PC, lysoPC and SM, significantly decreased after RHRT. Among amino acids, citrulline resulted the most altered metabolite (*p* = 9 × 10^−6^, *q* = 0.001), followed by taurine (*p* = 3 × 10^−5^, *q* = 0.002) and the acyl-carnitines C14 (*p* = 0.002, *q* = 0.04), C18:1 (*p* = 0.002, *q* = 0.05) and C18:2 (*p* = 2.8 × 10^−4^, *q* = 0.02), while for phospholipids, the significant changes regarded the PC ae C42:5 (*p* = 0.001, *q* = 0.03), PC ae C44:5 (*p* = 0.001, *q* = 0.03) and PC ae C44:6 (*p* = 3.7 × 10^−4^, *q* = 0.02) derivatives (Table 2). The individual concentration variations of such metabolites are shown in Figure S3 where it is possible to appreciate the homogeneous decreasing trend for each patient as a consequence of the RHRT treatment.

### 2.3. Metabolic Patterns Influenced by RHRT

**Figure 2:**
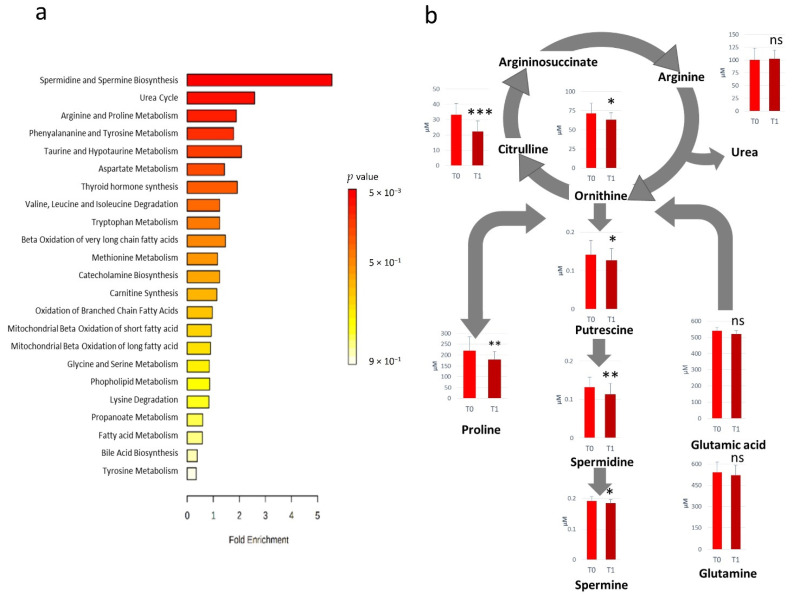
Over Representation Analysis plot from enrichment analysis. Bars represent matched pathways coloured according to their significance values, with gradations from yellow (low significance) to red (high significance) (**a**). Metabolic pathways altered as effect of RHRT and relative metabolites concentrations prior- (T_0_) and post-RHRT (T_1_) in 19 malignant pleural mesothelioma (MPM) patients. *p*-values derive from the Student’s *t*-test, *** *p* < 0.001, ** *p* < 0.01, * *p* < 0.05 (**b**).

All the metabolites significantly altered after RHRT were considered for the metabolic set enrichment analysis addressed to elucidate the biochemical pathways most influenced by RHRT. The Over Representation Analysis (ORA) indicates that polyamines biosynthesis, urea cycle as well as arginine and proline metabolism were the pathways more significantly perturbed by RHRT (Figure 2a). The polyamines biosynthesis pathway resulted downregulated, indeed the serum concentrations of putrescine, spermidine and spermine were significantly lower in post-RHRT serum samples (Figure 2b). Polyamines biosynthesis is linked to the urea cycle through ornithine whose serum level was found 12.3% lower post-RHRT (*p* = 0.02, *q* = 0.113). This latter amino acid is also the precursor of both citrulline and arginine. However, while arginine concentration remained constant and independent from the RHRT, citrulline underwent a dramatic drop (33%) (*p* = 9.0 × 10^−6^, *q* = 0.001). Such citrulline depletion was found highly correlated with that of ornithine (*r* = 0.72, *p* = 0.0005) but it was not associated with the common precursor glutamine, which did not undergo significant variations after RHRT. Analogously, proline, a further ornithine precursor, resulted in 19.2% lower post-RHRT compared with its baseline level (*p* = 5.3 × 10^−3^, *q* = 0.078).

### 2.4. Serum Metabolome Variations as Function of Radiation Dose

The amino acids class resulted mostly influenced by the RHRT since 14 out of 36 quantified amino acids and derivatives were significantly altered. Conversely, such widespread effect did not occur in the reference group subjected to palliative LRT at the dose of 21 Gy in 3 fractions. In this latter group, none of the metabolites included in the metabolomics profile analysis underwent significant variations, except citrulline that showed a slight (<10%) decrease (*p* = 0.04, *q* = 0.72) (Figure S4a). The mean fold changes of the metabolites belonging to the amino acids class in the two investigated groups are displayed in Figure S4b where it is evident that the RHRT produced higher variations on serum amino acid metabolites as compared with the LRT. The mean absolute variation of amino acids derivatives was 11.8% (range: 0.44–31.76%) and 4.0% (range: 0.26–11.8%) for RHRT and LRT treatments, respectively.

### 2.5. RHRT Metabolomics Alterations and Clinical Outcome

**Figure 3:**
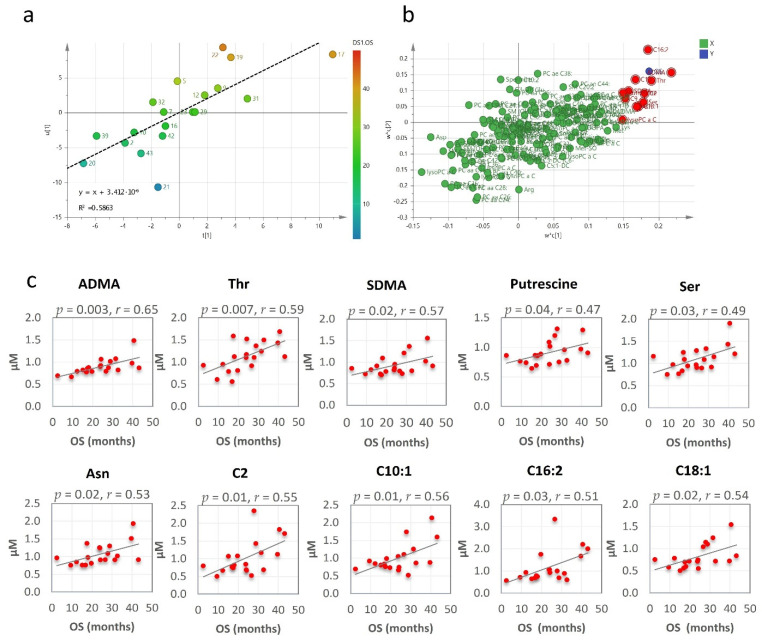
Partial least square (PLS) score plot for the first two latent variables t(1) and u(1), in which each point represents one patient, plotted as scores (or coefficients) from the metabolomics fold changes data (X block) vs. the score from the overall survival (OS) (Y block). Colour gradations from blue to red represents increasing values of OS (**a**). PLS loading plot. Each point is a metabolite plotted as the coefficient from PLS LV1 (first latent variable) vs. the coefficient from LV2 (second latent variable). Metabolites in the top right (highest positive coefficient) or in the bottom left (lowest negative coefficient) have a strong correlation with the OS (**b**). Metabolites fold changes most correlated with OS by Pearson correlation analysis (**c**).

The clinical outcome, expressed as median overall survival (OS), was 24 months (95% CI, 17–43 months) for the patients who underwent RHRT. At last follow-up, before the metabolomics data analysis, all investigated patients succumbed to the MPM disease. Their overall OS outcome was not found associated with age, tumour stage or performance status. Conversely, OS was significantly correlated with the serum metabolomics variations induced by RHRT. When partial least square (PLS) analysis was applied, the regression model showed a relevant association between the metabolites’ fold changes and the OS, which explained about 60% of interpatients OS variability (Figure 3a). The metabolites that most contributed to the model, as emerged by the loading plot (Figure 3b) and by Pearson correlation analysis were: asymmetric dimethyl-arginine (ADMA), threonine, symmetric dimethyl-arginine (SDMA), putrescine, serine, asparagine and the acyl-carnitines C2, C10:1, C16:2 and C18:1 whose serum variations were positively correlated with OS (Figure 3c).

**Figure 4:**
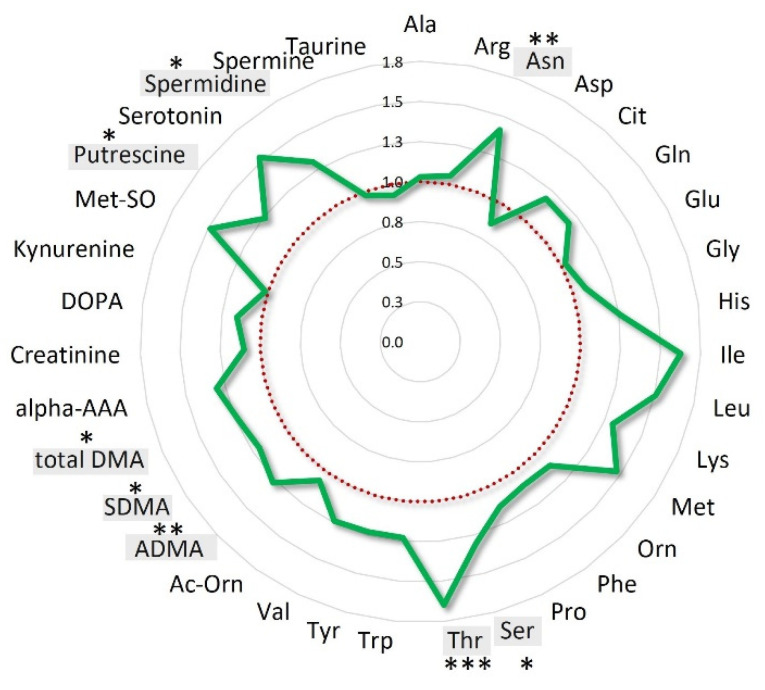
Amino acids mean overall variations of long survival patients normalized to those of short survival patients. Long survival patients belong to IQ (interquartile) and Q4 OS groups; short survival patients belong to the Q1 OS group. Amino acids statistically significant (*p* < 0.05) are highlighted in grey *** *p* < 0.001; ** *p* < 0.01, * *p* < 0.05.

When the patients’ population was stratified according to OS quartiles, the mean fold-changes of these specific metabolites were 0.77 ± 0.07 (range: 0.64–0.90) for the patients’ group with OS < 16.9 months (Q1), 1.01 ± 0.14 (range: 0.81–1.26) and 1.23 ± 0.17 (range: 0.98–1.47) for those with OS between 16.9 and 28.8 months (IQ) and > 28.8 months (Q4), respectively (Figure S5). Interestingly, when the analysis was focused on the entire class of amino acids, the overall variations for the long survival patients (16.9–43.17 months) resulted 22% higher than that observed for short survival patients (OS < 16.9) (Figure 4).

## 3. Discussion

The application of the ionizing radiation to tumour and surrounding normal tissues elicits complex responses that, behind the DNA cytotoxic activity, disrupt tumour metabolic processes and influence the overall host biochemistry [24].

The present study highlighted that in MPM patients the RHRT treatment is able to produce remarkable systemic metabolomics alterations involving a large set of biochemical pathways as a result of its activity on both normal and tumour tissues. This RT modality was found to produce an overall serum decrease in almost all investigated metabolites. Such depleting effect was commonly observed also for other RT treatment and cancer types suggesting that these serum metabolome drop phenomena could represent a distinctive tract of the host systemic metabolic response to irradiation [18,20,21,22,23,25]. Among the metabolites found altered after RHRT, only a small set that included valine and leucine was upregulated. These BCAAs are involved in protein metabolism, energy production and in various biosynthetic pathways which are all overactivated in tumour cells [26,27]. The high BCAA catabolism reported in tumour tissues has been found associated with a systemic deprivation of these amino acids [27,28,29]. In this context, the post-RHRT increase in valine and leucine may suggest a reduced tumour demand of BCAA as analogously observed in breast cancer patients where a specific raise of serum isoleucine, leucine and valine to normal range was reported after RT treatment [17]. Beyond BCAA, phenylalanine was another essential amino acid significantly increased post-RHRT. This amino acid increases during inflammation conditions [30,31] and its serum levels were found correlated with those of immune activation markers such as neopterin and isoprostane-8 [32,33]. The radiation exposure is known to promote oxidative stress leading to an acute inflammatory status [34,35] and mounting evidence indicates as the RT itself could also stimulate the immune system [36,37,38,39] that may be indirectly mirrored by the increase in serum phenylalanine observed after RHRT.

Notably, RHRT was found to influence extensively the lipid metabolism, and in particular, choline-containing phospholipids such as PC, lysoPC and SM derivatives, which underwent a significant serum concentration drop likely associated with the elevated lipids membrane turnover consequent to radiation tissue damage. Thus, this effect may not be specific for RHRT treatment but rather a common trait of the radiation exposure, since a wide lipids drop was also reported in other metabolomics studies regarding different RT treatments [18,19,22,40]. Phospholipids have not only a structural role in the cellular membrane but they also act as signal-transducer metabolites in different cellular pathways including apoptosis [41,42,43]. In this context, their downregulation, consequent to the RHRT treatment, may contribute to disrupt tumour signalling pathways and synergize with the radiation cellular killing effect [44]. Beyond phospholipids, the acyl-carnitines metabolite class was also found perturbed by RHRT. These lipids derivatives play a critical role in energy production working as a shuttle of fatty acids into the mitochondria, where they undergo β-oxidation for ATP production. In the investigated series of patients, the concomitant increase in carnitine precursor and the overall decrease in acyl-carnitines derivatives may suggest an alteration in their synthesis likely due to low availability of free fatty acids or acetyl-CoA intermediates that may be diverted to restore the phospholipids pool. Despite the broad perturbation of lipid metabolism, only amino acids-related pathways emerged from the set enrichment analysis. These involved the polyamine biosynthesis, urea cycle, and the arginine and proline metabolism that are strictly interconnected to each other sharing arginine as central metabolite. This latter is synthesized in the kidney and, besides its involvement in the urea cycle for ammonia detoxification, it is the substrate for other essential cellular metabolic pathways such as nitric oxide (NO) production [45]. The main endogenous source of arginine is citrulline that was the metabolite subjected to the highest decrease after RHRT. This nonproteogenic amino acid is synthesized in the small intestine from glutamine and ornithine precursors, but it can be also produced in other tissues as a recycled product of the NO synthesis [46]. The citrulline depletion after RHRT might be attributed to the reduction in its enterocytes biosynthesis as a side effect of the high-dose radiations that partially reach the hemithorax surrounding organs. Indeed, citrulline is a well-known biomarker of intestinal failure and a decrease in its blood concentration was registered in inflammatory bowel diseases [47] as well as in patients who received chemotherapy [14,48,49,50] or RT [51,52]. The systemic loss of citrulline does not seem to affect the arginine synthesis, since its level was unvaried by RHRT, suggesting that the host metabolism maintains a systemic reservoir of such semiessential amino acid at the expense of citrulline.

The RHRT effect is not limited only to arginine pathways but encompasses the whole class of amino acids likely consequent to the high dose of RHRT. Indeed, the patients treated with a low palliative dose of LRT did not show such significant alterations in the observed time-frame compared with those who received the high dose of RHRT. However, a modest but significant citrulline serum shortage was detected also in the LRT group where the involvement of the intestine was negligible suggesting other citrulline fates. Interestingly, citrulline has been revealed to exhibit antioxidant properties working as a suicidal radical-scavenger [53,54], thus in both RHRT and LRT groups, it may be oxidized by the reactive oxygen species (ROS) produced over the RT treatments. In addition, the radiolysis of the protein lysine-residuals leads to the release of alpha-aminoadipic acid [55,56] that was significantly high in the serum post-RHRT. Radiation oxidative injuries could be also suppressed by the sulphur amino acid taurine that works as antioxidant by reinforcing the endogenous radical-scavenger cellular systems [57,58,59,60] as recently demonstrated in lung tissues animal model [61]. Therefore, the taurine serum exhaustion after RHRT may indicate an increased tissue up-take of this amino acid as a consequence of the oxidative stress induced by the treatment.

Ornithine, another amino acid belonging to the arginine metabolism, was found significantly affected by RHRT treatment. Its serum decrease was found significantly correlated with that of citrulline being ornithine, together with glutamine, the principal precursor of citrulline [46]. Ornithine can be synthetized also from proline [62], which significantly decreased post-RHRT, suggesting that the radiation treatment can influence the whole ornithine biosynthetic pathways. The ornithine shortage may have relevant consequences because it represents an important precursor of putrescine, spermidine and spermine, collectively called polyamines. These cationic amino acid derivatives play a key role in cell proliferation [63] and the pharmacological inhibition of their synthesis has been demonstrated to induce tumour growth suppression in xenograft models of MPM [64]. In the context of this study, the significant downregulation of the polyamines, due to the low availability of their common precursor ornithine, may be translated into a potential inhibition of tumour growth with beneficial effect on MPM disease control.

Taken all together, these serum metabolomics changes seem to reflect the overall host metabolic mobilization not only to deal with the radiotoxic effects but also to indirectly control the MPM tumour growth. In agreement with this hypothesis, the individual metabolic response to RHRT could have implications in determining the patients’ clinical outcome. Indeed, a significant association between the metabolic profile variations and the OS was found for the amino acids and acyl-carnitines derivatives such as ADMA, threonine, SDMA, putrescine, asparagine and serine as well as the acylcarnitines C2, C10:1, C16:2 and C18:1. The patients’ groups with medium and high OS showed a higher increase in these metabolites after RHRT indicating that a greater metabolic response to RHRT may yield a better outcome. This observation can be extended to the whole amino acids metabolism further supporting that in long survival patients, the RHRT stimulates a highly dynamic metabolic response likely associated with a superior individual biochemical resilience. Such metabolic activity can be attributed to their high biological reserves availability that allows not only to better contrast the stress but also to integrate the potential stimulating effects of RHRT.

The low sample size of the present investigation does not allow to properly validate the results, and longitudinal studies with a larger cohort of patients are needed before the discovered systemic metabolomics signatures may find definitive clinical applications. Further investigations have to include time-series analyses along the RT treatment and the patients’ follow-up to distinguish the acute and long-term effects of RHRT on patients’ metabolomics profiles as well as to better identify the most powerful prognostic biomarkers. Moreover, the extension of the coverage of the metabolome considering other biological matrix such urine would allow having a full view of the metabolic response to RHRT.

## 4. Materials and Methods

### 4.1. Patients’ Population

This metabolomics study enrolled 34 patients from 2014 to 2018 with histologically confirmed MPM referred to Centro di Riferimento Oncologico of Aviano, Italy, for RT after nonradical surgery and systemic chemotherapy. All patients were enrolled within an ongoing randomized phase III study addressed to assess the OS advantages of RHRT over palliative LRT treatment. A test group of 19 patients received RHRT while a reference group of 15 patients underwent standard palliative LTR. The RHRT treatment was delivered with curative intent by IMRT technique to the hemithorax at the pleural surface level from the lung apex to the upper abdomen at the dose of 50 Gy in 25 fractions. The dose was delivered so that 95% of the planned target volume (PTV) was covered by 95% of the prescription dose. Tumour sites with high-fluorodeoxyglucose avidity received simultaneous integrated boosts of 60 Gy. The LRT for the reference group of patients was delivered at 21 Gy in 3 fractions at the thoracotomy scar level. All patients belonging to the test and the reference groups had good respiratory function and normal baseline renal, hepatic and bone marrow functions. The investigation was carried out in accordance with the principles of the Declaration of Helsinki and with approval from Ethics Committee of Centro di Riferimento Oncologico di Aviano (Clinical Trial code ID: CRO-2013–38). All subjects gave written informed consent. 

### 4.2. Sample Collection

Overnight fasting sample (5 mL) was collected from peripheral venous blood at the baseline and at the end of RHRT and LRT treatments. The blood was allowed to clot for 30 min at room temperature and then centrifuged at room temperature for 15 min at 2100 rpm. Serum samples were immediately stored at −80 °C until metabolomics analysis. 

### 4.3. Study Design

The study aims to explore the systemic metabolomics effects induced by RHRT in a group of 19 MPM patients. For each enrolled patient, serum targeted metabolomics profile was investigated both at the baseline, before the delivery of the RHRT and at the end of the daily 50 Gy/25 fractions administration. The significant metabolomics changes induced by such RT modality were analysed by univariate and multivariate analysis. The serum metabolomics alterations consequent to RHRT were compared with those of a reference group who received LRT to better distinguish the metabolic pathways specifically induced by RHRT.

### 4.4. Targeted Serum Metabolomics Profile Analysis

Metabolomics analysis of serum samples was performed using the Biocrates Absolute-IDQ P180 kit (Life Science AG, Innsbruck, Austria) targeted to 188 metabolites belonging to the following classes: amino acids (*n* = 21), biogenic amines and polyamines (*n* = 19), acylcarnitines (*n* = 40), lysophosphatidylcholines (*n* = 15), phosphatidylcholines (*n* = 77), sphingolipids (*n* = 15) and hexoses (*n* = 1). The list of all measured metabolites is reported in Table S1. Sample preparation was carried out following the manufacturer’s instructions. Briefly, after thawing, 10 µL of serum was transferred into a filter on the upper well of a 96-well sandwich plate. A mixture of internal standards labelled isotopically with deuterium, ^13^C or ^15^N was already present in each well. Nitrogen steam drying of filters was followed by derivatization of amino acids with 5% phenyl isothiocyanate (PTC) and a second drying step. Metabolites were then extracted with 500 µL of 5 mM ammonium acetate in methanol and the extraction solution was filtered and diluted with MS running solvent for the analysis.

The instrumentation consisted of a LC ultimate 3000 (Thermo Fisher Scientific, Milan, Italy) coupled with a 4000 QTAP (AB Sciex Framingham, MA, USA) mass spectrometer. Flow injection analysis coupled with tandem mass spectrometry (FIA-MS/MS) was used for the analysis of carnitine, acylcarnitines, lipids and hexoses, while liquid chromatography with tandem mass spectrometry (LC-MS/MS) was used for amino acids and biogenic amines PTC-derivatives separated in a ZORBAX SB 100 × 2.1 mm column (Agilent, Santa Clara, CA, USA). The triple quadruple operated in multiple reaction monitoring, neutral loss and precursor ion scan modes in positive and negative polarity. The MS/MS signals were integrated by Analyst 1.6.1 software (AB Sciex, Framingham, MA, USA) and quantified using a calibration curve according to the manufacturer’s instructions. Quality controls (QCs) at three concentration levels, low (QC1), medium (QC2) and high (QC3), were used to evaluate the performance of the analytical assay using the MetIQ software. Metabolites with serum concentration under the limit of detection were excluded for the statistical analysis.

### 4.5. Statistical Data Analysis

Quantitative metabolomics data were preprocessed by log transformation and autoscaling normalization. Unsupervised multivariate PCA of serum metabolomics data was applied to identify outliers. Supervised OPLS-DA was used to classify the metabolomics dataset and build a model able to differentiate serum metabolomics profiles at baseline (T_0_) and post-RHRT (T_1_). The OPLS-DA was validated to exclude data over-fitting using LOOCV by evaluation of the goodness of fit (R^2^) and predictive ability (Q^2^) values and by random permutation test to verify the true predictive ability of the model. Analysis of variance of cross-validated predictive residuals (CV-ANOVA) was computed for assessing model reliability. Variable Importance in Projection (VIP) that ranks the metabolites contribution in the OPLS-DA model and paired univariate Student’s *t*-test were used to identify metabolites whose concentrations differed significantly between T_0_ and T_1_. Multiple testing false discovery rate (FDR) correction was performed according to Benjamini–Hochberg method and a *q* < 0.05 was considered statistically significant unless otherwise specified.

Metabolite Set Enrichment Analysis was applied to detect the relevant metabolic pathways significantly altered by RHRT. All the metabolites selected by VIP > 1 and *p* < 0.05 were imported and matched in HMDB, PUBCHEM, SMPDB and KEGG databases, thus categorized according to SMPDB library. The most meaningful biochemical patterns altered by RHRT were inferred by the ORA plot where the metabolic pathways were ranked according to their significance values.

Association between the serum metabolomics fold-change (ratio T_1_/T_0_) and the OS of the patients, calculated from the date of first radiation fraction administration to the death date, was investigated by multivariate PLS analysis between two groups of variables. The Y variable (OS) was predicted using a few linear combinations of X variables (metabolites fold-changes) called latent variables (LVs). The extent of the correlation was evaluated by the regression coefficient of the PLS model (R^2^), while the validation of PLS regression model was performed by LOOCV and permutation test. The metabolites that best contributed to the PLS model were identified and selected by the loading plot for the component w*c [1] >0.15 and <−0.15. Data analysis and the statistical evaluations were carried out using SIMCA (Umetrics, v. 14.1) software, GraphPad Prism 7 and MetaboAnalyst v. 4.0 [65].

## 5. Conclusions

The results of this first exploratory study support the integration of metabolomics for the clinical evaluation of MPM patients. The metabolomics investigation may contribute to better understand the mechanisms underlying the interpatients OS variability to RHRT treatment and to recognize frail patients as a function of their specific metabolic phenotypes. Further validation of such powerful diagnostic tool could effectively improve the selection of the patients who could not receive clinical benefit from the RHRT treatment moving toward alternative personalized treatments.

## Figures and Tables

**Table 1:** Clinical characteristics of 19 malignant pleural mesothelioma (MPM) patients.

Characteristics	*n* (%)
Age (years), median, range	70 (33–79)
Sex	
Female	2 (11%)
Male	17 (89%)
Performance Status *	
0	6 (31%)
1	10 (53%)
2	3 (16%)
Histology	
Epithelioid	18 (95%)
Nonepithelioid	1 (5%)
Stage	
I–II	9 (47%)
III–IV	10 (53%)
Chemotherapy	
Pemetrexed, cisplatin	19 (100%)
Surgery	
Pleurectomy/decortication (P/D)	5 (26%)
Decortication	2 (11%)
Biopsy	12 (63%)

* Evaluated by ECOG, Eastern Cooperative Oncology Group.

**Table 2:** Metabolites significantly altered as effect of radical hemithoracic radiotherapy (RHRT) in 19 MPM patients.

Class	Name	Mean (µM) ± SD		Fold Change	Trend	*p-*Value	*q-*Value
		Baseline	Post-HRT				
Amino acids and derivatives	Cit	33.22 ± 7.02	22.19 ± 7.15	0.67	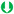	9.0 × 10^−6^	0.001
	ADMA	0.57 ± 0.06	0.5 ± 0.09	0.88	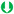	0.007	0.085
	Orn	71.74 ± 9.56	62.89 ± 13.15	0.88	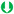	0.017	0.113
	Pro	220.95 ± 36.99	178.47 ± 63.76	0.81	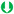	0.005	0.078
	Putrescine	0.14 ± 0.03	0.13 ± 0.04	0.89	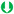	0.027	0.149
	Serotonin	0.48 ± 0.22	0.36 ± 0.22	0.75	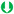	0.005	0.078
	Spermidine	0.13 ± 0.03	0.11 ± 0.03	0.86	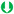	0.010	0.099
	Spermine	0.19 ± 0.01	0.18 ± 0.01	0.96	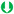	0.016	0.113
	Taurine	104.05 ± 18.69	74.49 ± 22.79	0.72	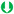	3.0 × 10^−5^	0.002
	total DMA	1.06 ± 0.31	0.94 ± 0.34	0.88	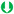	0.024	0.139
	Phe	70.96 ± 11.05	79.71 ± 8.17	1.14	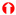	0.009	0.095
	Val	178.21 ± 38.35	202.63 ± 36.56	1.12	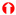	0.035	0.176
	alpha-AAA	1.17 ± 0.58	1.42 ± 0.34	1.21	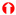	0.035	0.176
	Trp	46.96 ± 9.37	52.78 ± 10.89	1.12	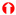	0.039	0.176
Acyl-carnitines	C10:2	0.08 ± 0.01	0.06 ± 0.04	0.73	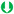	0.012	0.104
	C14	0.06 ± 0.02	0.04 ± 0.02	0.79	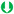	0.002	0.038
	C14:1-OH	0.02 ± 0.01	0.02 ± 0.01	0.83	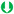	0.032	0.174
	C:16	0.15 ± 0.03	0.12 ± 0.05	0.81	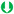	0.012	0.104
	C16:2-OH	0.01 ± 0.005	0.01 ± 0.005	0.81	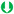	0.037	0.176
	C18:1	0.19 ± 0.04	0.14 ± 0.06	0.77	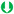	0.002	0.048
	C18:2	0.06 ± 0.02	0.04 ± 0.02	0.66	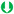	2.8 × 10^−4^	0.015
	C0	37.26 ± 6.11	41.23 ± 6.00	1.11	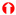	0.022	0.138
Phospholipids	lysoPC a C18:0	24.71 ± 6.45	20.01 ± 6.61	0.81	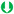	0.005	0.078
	PC aa C28:1	2.85 ± 0.84	2.43 ± 0.95	0.85	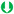	0.007	0.085
	PC aa C36:2	203.53 ± 51.70	173.58 ± 42.35	0.85	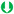	0.012	0.104
	PC aa C38:0	3.04 ± 1.00	2.44 ± 1.58	0.8	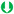	0.024	0.139
	PC aa C40:2	0.29 ± 0.11	0.22 ± 0.12	0.76	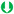	0.043	0.185
	PC ae C36:3	6.23 ± 1.31	4.94 ± 2.11	0.79	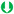	0.036	0.176
	PC ae C38:6	6.95 ± 2.27	5.92 ± 3.08	0.85	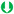	0.046	0.195
	PC ae C40:1	1.38 ± 0.42	1.14 ± 0.60	0.82	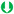	0.015	0.113
	PC ae C40:4	2.22 ± 0.32	1.77 ± 0.68	0.8	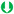	0.015	0.113
	PC ae C42:3	0.74 ± 0.23	0.61 ± 0.29	0.83	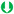	0.039	0.176
	PC ae C42:4	0.71 ± 0.25	0.47 ± 0.41	0.67	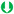	0.007	0.085
	PC ae C42:5	2.33 ± 0.30	1.67 ± 0.92	0.72	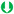	0.001	0.026
	PC ae C44:5	2.1 ± 0.42	1.37 ± 1.22	0.65	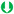	0.001	0.026
	PC ae C44:6	1.25 ± 0.31	0.85 ± 0.65	0.68	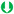	3.7 × 10^−4^	0.015
Sphyngomielyns	SM C24:0	18.29 ± 5.87	14.75 ± 4.30	0.81	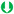	0.018	0.113
	SM C24:1	48.12 ± 13.66	41.99 ± 11.09	0.87	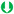	0.047	0.195
	SM-OH C24:1	1.25 ± 0.3	0.93 ± 0.49	0.74	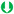	0.014	0.113

Fold change, metabolite concentration after RHRT divided by baseline concentration; Cit, citrulline; Orn, ornithine; Pro, proline; Phe, phenylalanine; Val, valine; alpha-AAA, alpha-amino adipic acid; Trp, tryptophan; Cn:z, acylcarnitine, *n* = number of carbons, *z* = number of unsaturations; lysoPC Cn:z, lysophosphatidylcholine; PC Cn:z, phosphatidylcholine; SM Cn:z, sphingomyelins; SM OH Cn:z, hydroxylated sphingomyelins. In bold are metabolites with *q*-values < 0.05. 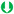, down-regulated; 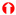, up-regulated.

## Data Availability

The data presented in this study are available on request from the corresponding author.

## Supplementary Materials

The following are available online at https://www.mdpi.com/2072-6694/13/3/508/s1, Figure S1: Principal component analysis (PCA) of metabolomics profiles of 19 malignant pleural mesothelioma (MPM) patients before (T_0_) and after (T_1_) radical hemithoracic radiotherapy (RHRT), Figure S2: Metabolites ranked according to their Variable Importance in Projection (VIP) scores in the orthogonal partial least squares discriminant analysis (OPLS-DA) model, Figure S3: Serum concentrations at baseline (T_0_) and post-RHRT (T_1_) for metabolites resulted significantly altered in 19 MPM patients, Figure S4: Citrulline serum concentrations at baseline (T_0_) and post-RHRT (T_1_) in MPM patients underwent local radiotherapy (LRT) and RHRT, Figure S5: Fold changes of serum amino acids and derivatives expressed in MPM patients under RHRT and LRT treatments, Table S1: Metabolites included in the targeted metabolomics analysis, Table S2: Clinical characteristics of LRT group’s patients.
