# Supplementary material for: Radical Hemithoracic Radiotherapy Induces Systemic Metabolomics Changes That Are Associated with the Clinical Outcome of Malignant Pleural Mesothelioma Patients
Source: Cancers (Basel). 2021 Jan 29;13(3):508. doi: 10.3390/cancers13030508 (PMC7866164; doi:10.3390/cancers13030508)
Supplement: Supplementary file 1 [file cancers-13-00508-s001.pdf]

cancers

MDPI

Supplementary Materials: Radical Hemithoracic Radiotherapy Induces Systemic Metabolomics Changes That Are Associated With The Clinical Outcome Of Malignant Pleural Mesothelioma Patients

Emanuela Di Gregorio, Gianmaria Miolo, Asia Saorin, Elena Muraro, Michela Cangemi, Alberto Revelant, Emilio Minatel, Marco Trovò, Agostino Steffan and Giuseppe Corona

Citation: Di Gregorio, E.; Miolo, G.; Saorin, A.; Muraro, E.; Cangemi, M.; Revelant, A.; Minatel, E.; Trovò, M.; Steffan, A.; Corona, G. Radical Hemithoracic Radiotherapy Induces Systemic Metabolomics Changes That Are Associated with the Clinical Outcome of Malignant Pleural Mesothelioma Patients. *Cancers* **2021**, *13*, x. https://doi.org/10.3390/xxxxx

Academic Editor: Daniel L. Pouliquen, Joanna Kopecka
Received: 23 December 2020
Accepted: 25 January 2021
Published: 29 January 2021

Publisher's Note: MDPI stays neutral with regard to jurisdictional claims in published maps and institutional affiliations.

Copyright: © 2021 by the authors. Licensee MDPI, Basel, Switzerland. This article is an open access article distributed under the terms and conditions of the Creative Commons Attribution (CC BY) license (http://creativecommons.org/licenses/by/4.0/).

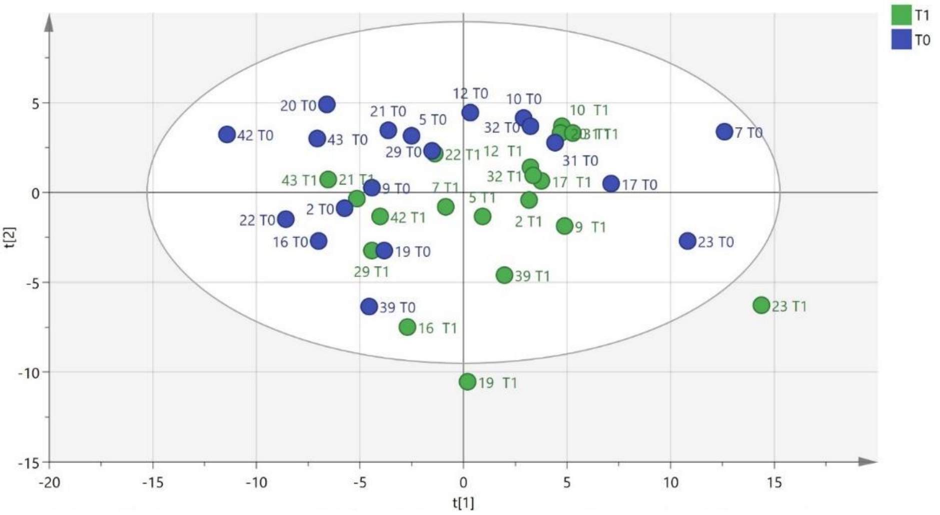

Figure S1. PCA of metabolomics profiles of 19 MPM patients before (T_0) and after (T_1) RHRT.

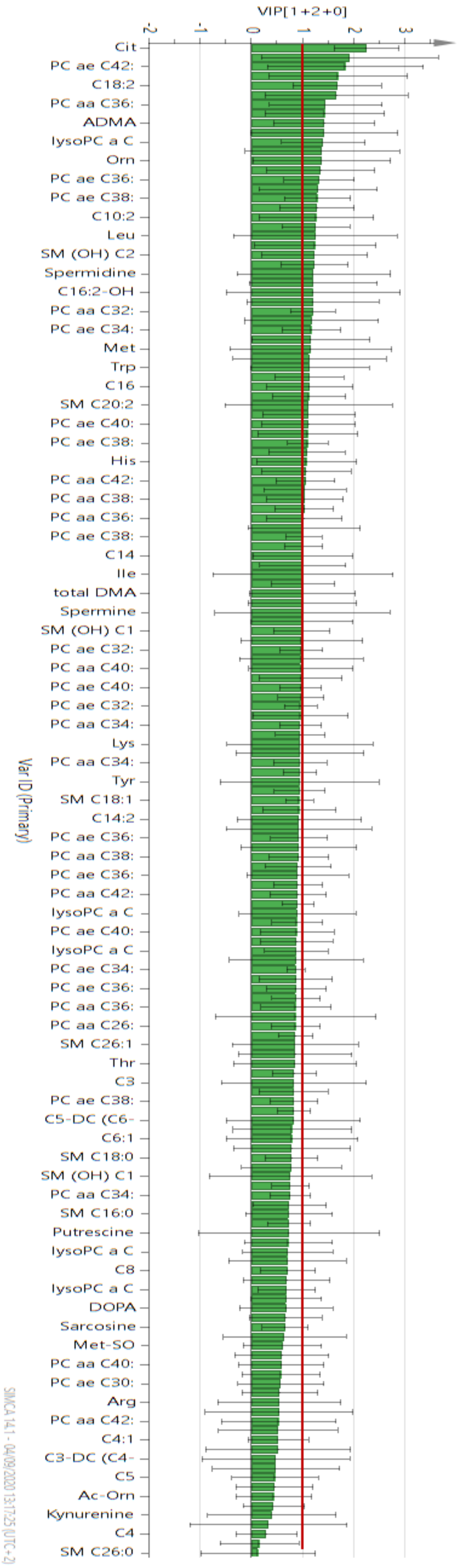

Figure S2. Metabolites ranked according to their VIP scores in the OPLS-DA model. The bars in the histograms are the confidence intervals derived from jack-knifing.

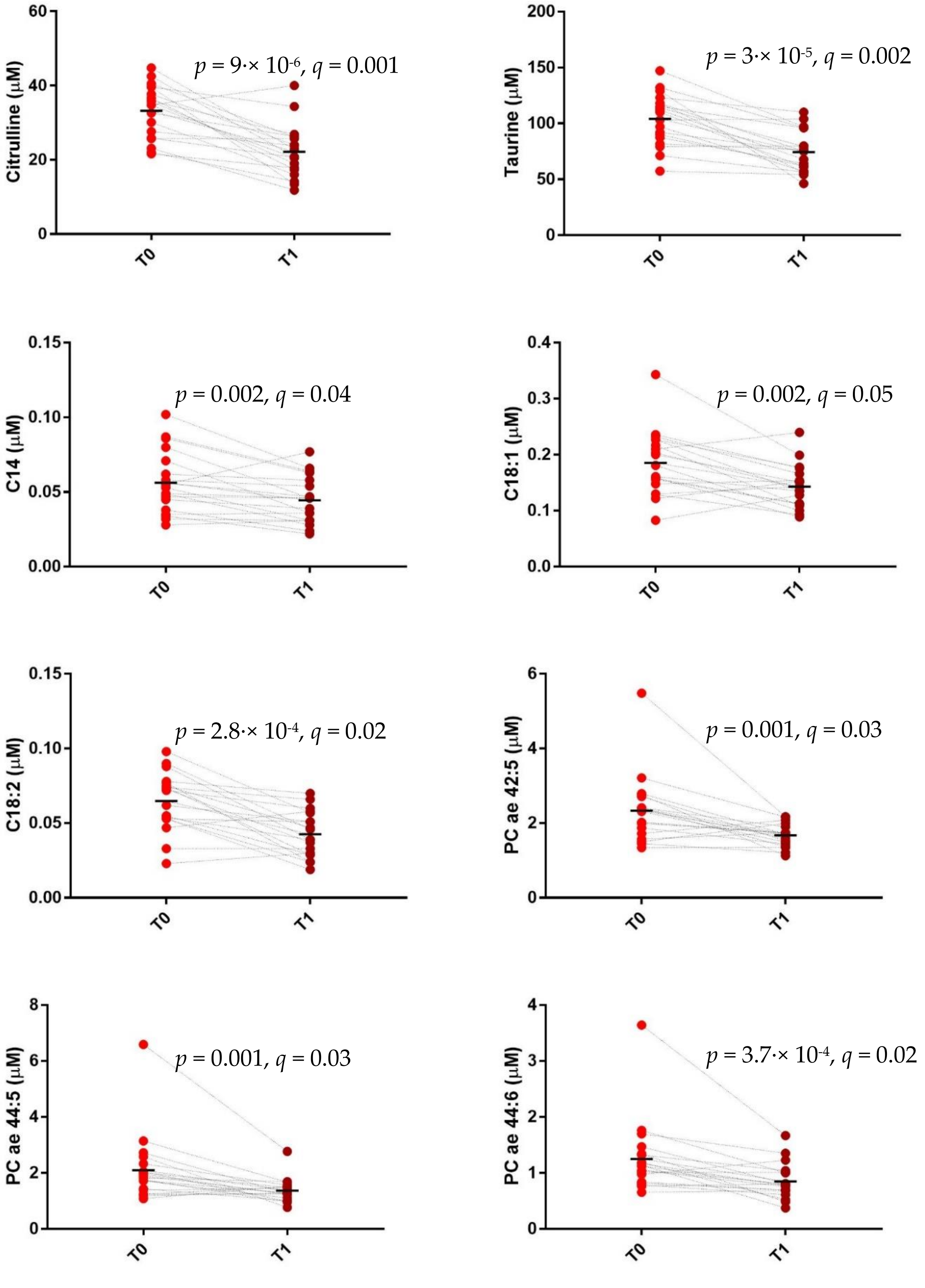

Figure S3. Serum concentrations at baseline (T_0) and post-RHRT (T_1) for metabolites resulted significantly altered ($q < 0.05$, Student's *t*-test) in each MPM patient ($n = 19$) underwent RHRT. The black line indicates the average value for both time points.

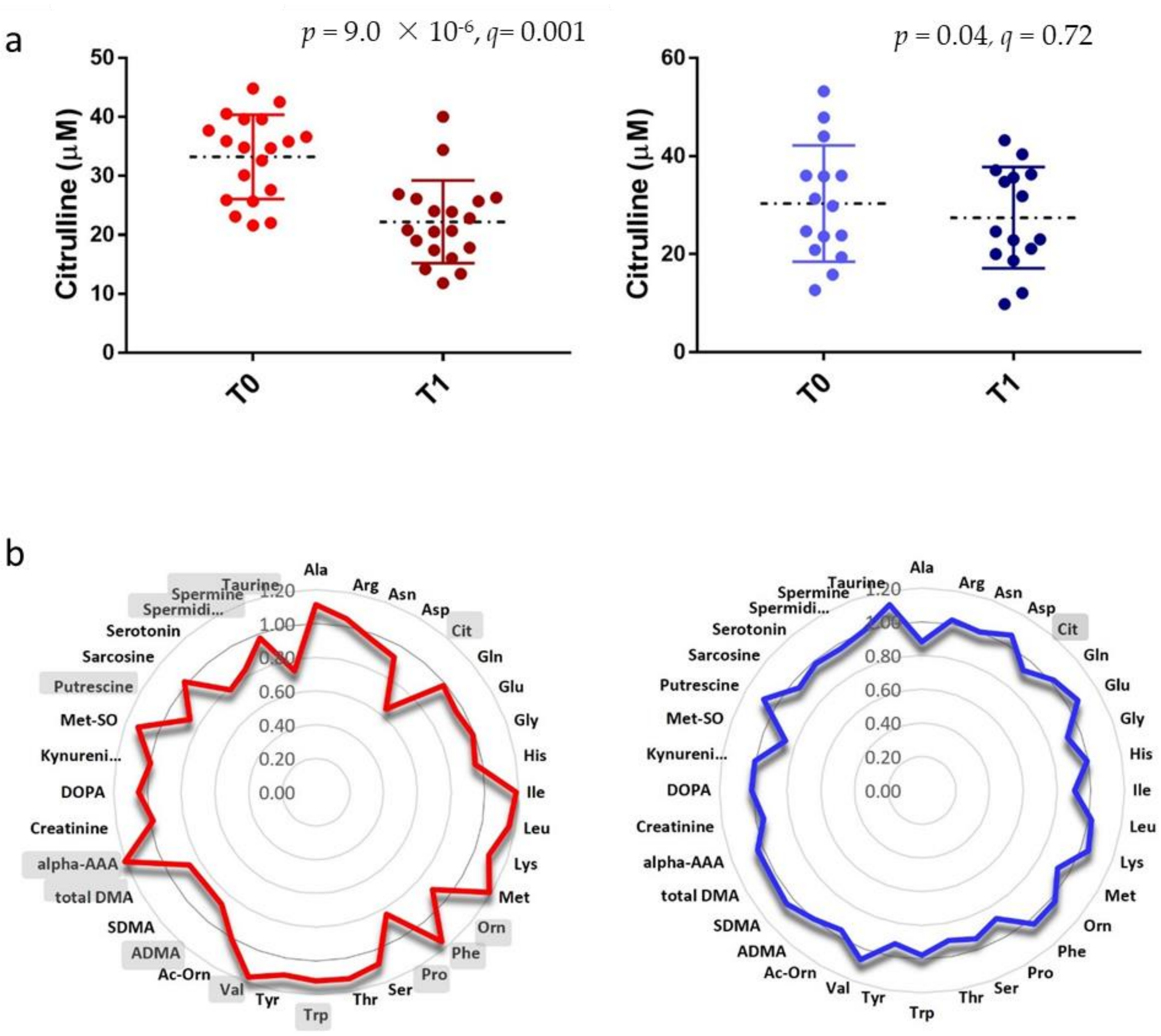

Figure S4. Citrulline serum concentrations at baseline (T_0) and post-RHRT (T_1) in MPM patients underwent RHRT (left) and LRT (right) **(a)**. Mean fold changes of serum amino acids and derivatives in MPM patients under RHRT treatment (red, n = 19) and under LRT (blue, n = 15). Metabolites significantly changed in patients underwent RHRT are highlighted in grey **(b)**.

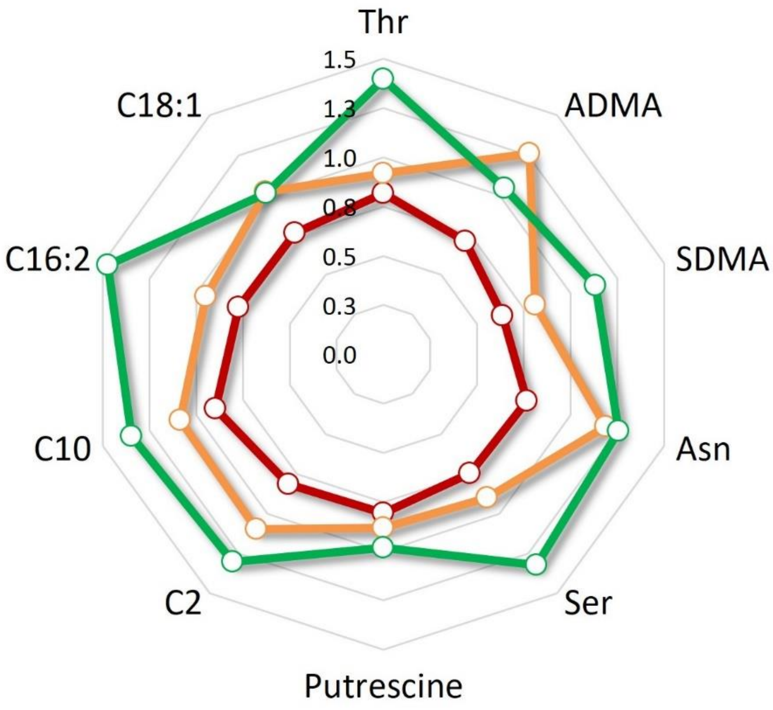

Figure S5. Mean fold change (ratio T_1/T_0) of metabolites correlated with OS in patients classified in quartiles according to OS. Short survival patients with low OS belonging to Q1 (red) had a mean fold change <1, conversely patients of IQ (orange) with medium OS and patients of Q4 (green) with high OS showed a mean fold change ≥1 (p = 0.001).

Table S1. Metabolites included in the targeted metabolomics analysis.

Metabolites Class	*n*	Abbreviations
Amino acids	21	Ala,Arg,Asn,Asp,Cit,Gln,Glu,Gly,His,Ile,Leu, Lys,Met,Orn,Phe,Pro,Ser,Thr,Trp,Tyr,Val.
Biogenic amines	19	ADMA, AcOrn, carnosine, creatinine, histamine, kynurenine, Met-SO ,Nitro-Tyr, OHPro,PEA,Putrescine,SDMA,sarcosine,seroto ni n,spermidine,spermine,taurine,alphaAAA,tot alDMA.
Carnitine	1	C0
Acylcarnitines	39	C2, C3, C3:1, C4, C4:1, C5, C5:1, C6 (or C4:1-DC), C6:1, C8, C8:1, C9, C10, C10:1, C10:2, C12, C12:1,C14, C14:1, C14:2, C16, C16:1, C16:2, C18, C18:1,C18:2, C3-OH, C4-OH (or C3-DC), C5-DC (or C6- OH), C5-OH (or C3-DC-M), C5:1-DC, C5-MDC, C7-DC, C12-DC, C14:1-OH, C14:2-OH, C16:1-OH, C16:2-OH, C16-OH, C18:1-OH.
Hexoses	1	H1
Sphingomyelins	15	SM 16:0, SM 16:1, SM 18:0, SM 18:1, SM 20:2, SM 22:3, SM 24:0, SM 24:1, SM 26:0, SM 26:1,

		SM (OH) 14:1, SM (OH) 16:1, SM (OH) 22:1, SM(OH) 22:2, SM (OH) 24:1.
Diacyl-phosphatidyl-cholines	38	PC aa 24:0/26:0/28:1/30:0/30:2/32:0/32:1/32:2/32:3/34:1/34:2/34:3/34:4/36:0/36:1/36:2/36:3/36:4/C36:5/36:6/38:0/38:1/38:3/38:4/38:5/38:6/40:1/40:2/40:3/40:4/40:5/40:6/42:0/42:1/42:2/42:4/42:5/42:6.
Acyl-alkylphosphati-dylcholines	39	PC ae 30:0/30:1/30:2/32:1/32:2/34:0/34:1/34:2/34:3/C36:0/C36:1/36:2/36:3/36:4/36:5/38:0/38:1/38:2/38:3/38:4/38:5/38:6/40:0/40:1/40:2/40:3/40:4/40:5/40:6/42:0/42:1/42:2/42:3/42:4/42:5/44:3/44:4/44:5/44:6.
Lyso-phosphatidyl-cholines	15	LysoPC aa 6:0/14:0/16:0/16:1/17:0/18:0/18:1/18:2/20:3/20:4/24:0/26:0/26:1/28:0/28:1.

Table S2. Clinical characteristics of LRT patients' group (n=15).

**Characteristics**	***n (%)***
Age (years), median, range	74 (51–80)
Sex	
Female	0
Male	15 (100%)
Performance Status *	
0	6 (40%)
1	7 (47%)
2	2 (13%)
Histology	
Epithelioid	11 (73%)
Non-epithelioid	4 (27%)
Stage	
I-II	6 (40%)
III-IV	9 (60%)
Chemotherapy	
Pemetrexed, cisplatin	15 (100%)
Surgery	
Pleurectomy/Decortication (P/D)	4 (27%)
Decortication	1 (6%)
Biopsy	10 (67%)

* Evaluated by ECOG, Eastern Cooperative Oncology Group.
